# Journal of molecular modeling conversations

**DOI:** 10.1007/s00894-022-05191-y

**Published:** 2022-08-25

**Authors:** Tim Clark

**Affiliations:** Computer-Chemie, Centrum Universität Erlangen-Nürnberg Nägelsbachstraße, 25, 91052 Erlangen, Germany

I am pleased to announce that the first *Journal of Molecular Modeling Conversation* is now ready for publication and will appear as consecutive contributions in both the online and print versions of the journal.

This Conversation on *Non-Covalent Interactions* is the first in a new series of special-format Topical Collections to be published in the Journal of Molecular Modeling. The idea for the conversations arose at a Dinner in Arnaud’s Restaurant in New Orleans attended by, among others Jane Murray, Paul Ayers, Rod Bartlett, Mel Levy, Peter Politzer, Kevin Riley and myself. Each *Conversation* will initially consist of typically four thought-provoking (and hopefully controversial) essays on current aspects of the general subject, in this case non-covalent interactions. Each of these essays is circulated among the authors, who add comments and questions that are subsequently answered. This initial Conversation is then published as a Topical Collection with the essays, comments, questions and answers. The format of the Conversations is as follows:

- Each *Conversation* is moderated by a *guest editor*, who invites typically four or five experts on different aspects of the subject of the *Conversation* to act as *primary contributors* by writing articles on their specific interest and expounding their views. The guest editor also writes a *primary contribution.*
- The *primary contributors* submit their contributions first to the *guest editor*, who circulates them among all *primary contributors*, who can pose written questions to be answered by the authors of the articles in question. This process can be iterated until the original articles, questions and answers are submitted all together to the Journal’s editorial system.
- After refereeing, the articles are revised as necessary so that they can appear back-to-back in the printed version of the journal and with consecutive paper numbers online.
- The *Conversation* appears as a Topical Collection that is initially open access. The editor in chief, *guest editor* and *primary contributors* can then invite others to contribute to the conversation, which will remain open for submissions for an agreed time after the primary articles appear. Interested readers can also submit comments and articles spontaneously. These contributions will be handled by the *guest editor*. Questions and answers will be solicited by the *guest editor* for manuscripts submitted in this second round of submissions and will be published in the same way as for the *primary contributions.*

That, however, is only the beginning. Once the initial essays become available, experts in the field can submit their own views in essays of their own. These will also be submitted to comments and questions, so that a true conversation in print results. There is no deadline for the end of the Conversation; it will be closed when the discussion ebbs.

The first *Journal of Molecular Modeling Conversation* has been edited by Tore Brinck on the topic of *Non-Covalent Interactions*. It features contributions from Tore himself, together with André Nyberg Borrfors, Krzysztof Szalewicz and Bogumil Jeziorski, Yirong Mo, David Danovich and Sason Shaik and by Paul Popelier with discussion contributions from all the authors. The Conversation is now open to opinions from all interested scientists. Just be sure to assign your submission to the “***TC Conversation***” We anticipate that the *Conversation* will be open for further contributions until June 1^st^ 2023 and look forward to stimulating discussions.

Tim Clark

Editor in Chief

31^st^ May 2023.

